# Decisional conflict in women with a *BRCA1* or *BRCA2* pathogenic variant who have not elected for risk-reducing salpingo-oophorectomy

**DOI:** 10.1186/s13053-025-00329-4

**Published:** 2026-01-17

**Authors:** Kelly A. Metcalfe, Anita Y. Kinney, Steven A. Narod, Aletta Poll, Susan Armel, Lucia Lombardi, Farideh Tavangar, Tuya Pal

**Affiliations:** 1 Lawrence S. Bloomberg Faculty of Nursing, University of Toronto, 155 College Street, Toronto, ON M5T 1P8 Canada; 2 Women’s College Research Institute, Toronto, Canada; 3 Rutgers Cancer Institute of New Jersey, New Brunswick, NJ USA; 4 Department of Biostatistics and Epidemiology, School of Public Health, Rutgers University, Piscataway, NJ USA; 5 Princess Margaret Cancer Centre, Toronto, Canada; 6 Department of Molecular Genetics, University of Toronto, Toronto, Canada; 7 Department of Psychological, Health and Territorial Sciences, University “G. d’Annunzio” of Chieti-Pescara, Chieti, Italy; 8 Vanderbilt-Ingram Cancer Center at the Vanderbilt University Medical Center, Nashville, TN USA

**Keywords:** BRCA1, BRCA2, Ovarian cancer, Risk, Decisional conflict, Distress, Bilateral salpingo-oophorectomy

## Abstract

**Objective:**

To identify predictors of decisional conflict among women with a *BRCA* pathogenic variant (PV) who were eligible for risk reducing salpingo-oophorectomy (RRSO) who had not made a decision to have surgery at least one year after receiving genetic test results.

**Methods:**

Women with a *BRCA1* or *BRCA2* PV between the ages of 35 and 70 years old, who had not elected for RRSO at least 12 months after receipt of genetic test results, were administered self-report questionnaires investigating demographic variables, decisional conflict (Decisional Conflict Scale), cancer-related distress (Impact of Event Scale) and cancer risk perception. Decisional conflict scores were generated and a multivariable linear regression was conducted to identify variables associated with decisional conflict.

**Results:**

A sample of 107 women completed questionnaires. Overall, 44 participants (41%) had a high decisional conflict score (greater than 37.5) related to the RRSO decision. Higher education (β = 11.40, 95% C.I: 0.59, 22.20; *p* = 0.039), non-white race (β = 11.12, 95% C.I: 0.66, 21.57; *p* = 0.037), and having children (β = 22.89, 95% C.I: 10.07, 35.71; *p* < 0.001) were significantly associated with higher decisional conflict. Lower decisional conflict was significantly associated with genetic testing more than 3 years prior (β = -13.14, 95% C.I: -23.27, -2.99; *p* = 0.012).

**Conclusions:**

Many women with a *BRCA* PV who have not elected for RRSO are experiencing high levels of decisional conflict related to the decision regarding RRSO. Interventions that target decisional conflict are needed to increase the uptake of RRSO which will result in a reduction of the risk of ovarian cancer in women with *BRCA1* or *BRCA2* PV.

## Introduction

Genetic testing to identify pathogenic variants (PV) in inherited cancer predisposition genes provides the opportunity to identify women who are at high-risk of developing cancer, and adopt cancer risk reduction strategies to prevent cancers from developing. Some of the highest known risks of ovarian cancer are in women with a *BRCA1* or *BRCA2* PV. Women with a *BRCA1* PV have an estimated 44% lifetime risk of developing ovarian cancer, and women with a *BRCA2* PV have a ~ 17% risk of ovarian cancer [1]. If a woman with a *BRCA* PV is diagnosed with ovarian cancer, it is typically diagnosed at an advanced stage when the cancer has spread beyond the ovary, and the ten-year survival rate is only 35% [2].

Screening for ovarian cancer in women with a BRCA1 or BRCA2 is ineffective [3]. Therefore, risk reduction surgery is offered to these high-risk women. The National Comprehensive Cancer Network (NCCN) recommends risk-reducing salpingo-oophorectomy (RRSO) (removal of the ovaries and fallopian tubes) between the ages of 35 and 40 years for *BRCA1* and 40 to 45 years for *BRCA2* [4]. This surgery reduces the risk of ovarian/peritoneal/fallopian tube cancer by 80% in women with a *BRCA* PV, and reduces the risk of all-cause mortality by 77% [2, 5].

We have previously reported on uptake of RRSO in an international sample of women with a *BRCA1* or *BRCA2* PV [6]. Overall, the uptake of RRSO was 65%, which is low considering that RRSO is recommended for women with a *BRCA* PV. Some women may have difficulty with decision making related to RRSO. Decisional conflict is indicative of the level of comfort an individual feels in making a decision [7], and decisional conflict scores predict the extent to which individuals make health-related decisions [8].

We have previously reported that a theory-based behavioural intervention delivered by genetic counsellors reduced decisional conflict related to RRSO at one-year, and higher uptake rates of RRSO at two-years [9]. It is therefore important to evaluate if there are factors that predict high decisional conflict in women with a *BRCA* PV faced with the decision regarding RRSO. Interventions, including the theory-based behavioural intervention [9], could then be targeted at women who may be at the highest risk of experiencing decisional conflict. By reducing decisional conflict, uptake of RRSO may increase in this high-risk population.

In the current study, we report on factors associated with decisional conflict in women with a *BRCA1* or *BRCA2* PV who had not elected for RRSO at least one year after receipt of genetic test results.

## Methods

### Study design

This study is a component of a larger randomized controlled trial that has previously been reported [9]. In the current study, a cross-sectional design was used in which data was collected from eligible participants prior to randomization for the parent randomized controlled trial. Eligible participants were identified in the clinic databases at the participating centres and contacted by telephone. The research assistant explained the study and consent was obtained. Participants completed a baseline questionnaire online (REDCap) or a hard copy was mailed if requested. The study was approved by Research Ethics Boards at all participating centres.

### Study participants

Eligible study participants included women enrolled in a multicenter, longitudinal study of *BRCA1* and *BRCA2* mutation carriers at five sites (Women’s College Hospital – Toronto, Ontario (ON); Grandriver Hospital, Kitchener, ON; London Health Sciences Centre, London, ON; Princess Margaret Cancer Centre, Toronto, ON; Moffitt Cancer Center, Tampa, FL) in addition to self-referrals through advertisements with commercial genetic testing laboratories and an online support group (Facing Our Risk of Cancer Empowered - FORCE). Ethics approval was obtained at all participating sites.

Eligibility criteria included the following: (1) documented *BRCA1* or *BRCA2* PV; (2) age 35 to 70 years; (3) no previous RRSO; (4) no previous or current ovarian cancer; (4) at least 12 months since genetic testing or most recent contact by parent follow-up study; (5) provided written consent to be re-contacted for future research; and (6) could speak and understand English.

Women were excluded if: (1) currently receiving treatment for another cancer diagnosis (including breast cancer); (2) pregnant; (3) given birth in the last 6 months; or (4) were scheduled for or already had a surgical date for RRSO.

### Measures

***Demographic and clinical characteristics.*** Participants were asked about age (in years), marital status (married/common law vs. divorced/separated/single), education level (university degree vs. none), household income (less than $80,000 vs. $80,000 or more), cultural group (non-Hispanic white vs. non-white) having children (yes, no) and planning to have (more) children (yes, no) at baseline survey.

Clinical characteristics were collected in the baseline survey and included having a sister or mother with an ovarian cancer diagnosis (yes, no), *BRCA* mutation type (*BRCA1*, *BRCA2*), annual ovarian screening (yes, no), time since genetic testing (months), perceived risk of ovarian cancer (between 0 and 100%), and planning on RRSO in the future.

***Decisional conflict*** was assessed using the *Decisional Conflict Scale (DCS).* [10] The DCS is a validated measure of decisional conflict and measures a person’s perception of the difficulty of making a decision (in this case about RRSO). The questionnaire consists of 16 items with response options ranging from 0 (strongly agree) to 4 (strongly disagree). Item responses are summed and scaled to create a total score ranging from 0 (no decisional conflict) to 100 (extremely high level of decisional conflict). Scores lower than 25 are associated with implementing decisions and scores exceeding 37.5 are associated with delay or feeling unsure about implementation [11].

***Cancer-related distress*** was assessed using the *Impact of Event Scale (IES)* [12], a self-report measure designed to measure current subjective distress about a specific stressor, in this case, risk of cancer. The scale consists of 15 items: 7 intrusion items and 8 avoidance items. Participants rate the frequency of intrusive and avoidant behaviours using a four-point frequency scale (0 = not at all, 1 = rarely, 3 = sometimes, 5 = often). Scores range from 0 to 75, with separate intrusion and avoidance sub-scales scores (with a possible range of 0 to 35 for intrusion, and 0 to 40 for avoidance). Lower scores depict lower levels of distress.

### Statistical analysis

Data analysis was performed using R version 4.3.1 software. Descriptive statistics were generated using means with standard deviations (SD) for continuous variables and frequency counts/percentages for categorical variables. Differences in the mean DCS scores at each variable level were tested using a Mann-Whitney U test due to non-normality of this score.

Multivariable linear regression was conducted to identify variables associated with decisional conflict. Continuous predictors included age and IES score; binary predictors included education level, marital status, household income, cultural group, having children, planning to have children, *BRCA* mutation, family history of ovarian cancer, annual ovarian screening, time since genetic testing in months, perceived risk of ovarian cancer, and planning RRSO in the future. We conducted regression diagnostics to ensure the adequacy of our model specification. This process involved assessing multicollinearity using the Variance Inflation Factor (VIF) to confirm that none of the variables had a VIF value exceeding 5. Additionally, we examined the normality of residuals. We also provided unstandardized regression coefficients along with their corresponding 95% confidence intervals.

## Results

A cohort of 107 women (mean of age 45.2 years) who had not undergone RRSO at least one year after receiving genetic test results were included in the study. Most participants held a university degree (63.6%), were married or common-law (71.7%), reported an annual household income of $80,000 or more (67.4%), self-identified as White (72.9%) and had children (77.6%).

Over half of the participants had a *BRCA1* pathogenic variant (57.0%), and 76.9% underwent genetic testing more than three years previously. The majority of participants reported having annual ovarian screening (57.9%), planned on having RRSO in the future (69.2%), and reported a 25% or greater perceived risk of ovarian cancer (78.5%). Detailed demographic and clinical variables are presented in Table 1.

**Table 1:** Demographic and clinical characteristics of sample, *n* = 107

Characteristic	*n* (%)
**Age**,** mean (SD)**	45.2 (8.5)
**Education**	
No university degree	39 (36.4)
University degree	68 (63.6)
**Marital Status**	
Single/ Divorced/separated	30 (28.3)
Married/common-law	76 (71.7)
**Household income**	
Less than $80,000	28 (32.6)
$80,000 or more	58 (67.4)
**Race**	
White non-Hispanic	78 (72.9)
Race other than White	29 (27.1)
**Have children**	83 (77.6)
**Planning to have children**	11 (10.3)
***BRCA*** **pathogenic variant**	
* BRCA1*	61 (57.0)
* BRCA2*	46 (43.0)
**Family history ovarian cancer**	23 (21.5)
**Undergo annual ovarian screening**	62 (57.9)
**IES score**,** mean (SD)**	17.8 (16.9)
**Time since genetic testing (months)**	
* ≤* 36	24 (23.1)
> 36	80 (76.9)
**Perceived risk of ovarian cancer**	
<25%	23 (21.5)
* ≥* 25%	84 (78.5)
**Planning RRSO in the future**	
No Yes	33 (30.8) 74 (69.2)

Table 2 presents the mean DCS scores by clinical and demographic variables. Total DCS scores ranged from 0 to 85.9 with a mean of 35.4 (SD 20.5). Overall, 28 women (26%) had scores less than 25 on the DCS and 44 women (41%) had scores greater than 37.5. Variables associated with decisional conflict are presented in Table 3. A significant linear regression model was found (F (14, 67) = 1.86, adjusted R-square = 12.0%, *p* = 0.044) indicating the hypothesized variables explained 12.0% of the variation in decisional conflict. The adequacy of the model specification was checked, and all regression model assumptions were met. Among demographic variables, holding a university degree (β = 11.40, 95% C.I: 0.59, 22.20; *p* = 0.039), non-White race (β = 11.12, 95% C.I: 0.66, 21.57; *p* = 0.037), and having children (β = 22.89, 95% C.I: 10.07, 35.71; *p* < 0.001) were significantly associated with higher levels of decisional conflict. Genetic testing more than 3 years prior was the only clinical variable significantly associated with a lower DCS score (β = -13.14, 95% C.I: -23.27, -2.99; *p* = 0.012). Family history of ovarian cancer, undergoing annual ovarian screening, and total IES score were not found to be associated with decisional conflict.

**Table 2:** Decisional conflict scores by demographic and clinical characteristics, *n* = 107

Characteristics	Mean (SD)	*P*-value
**Age**		0.11
<40 years	40.3 (20.8)	
*≥*40 years	33.7 (20.3)	
**Education**		0.89
No university degree	37.1 (23.4)	
University degree	34.4 (18.8)	
**Marital Status**		0.73
Single/ Divorced/separated	35.1 (15.8)	
Married/common-law	35.0 (21.8)	
**Household income**		0.25
Less than $80,000	37.4 (19.6)	
$80,000 or more	32.5 (19.6)	
**Race**		0.20
White non-Hispanic	34.1 (20.5)	
Non-White	38.7 (20.7)	
**Have children**		0.10
No	28.1 (15.8)	
Yes	37.5 (21.3)	
**Planning to have children**		0.11
No	34.2 (19.8)	
Yes	45.6 (24.9)	
**BRCA pathogenic variant**		0.44
* BRCA1*	36.9 (20.8)	
*BRCA2*	33.4 (20.2)	
**Family History Ovarian Cancer**		0.34
**No**	34.9 (21.8)	
**Yes**	37.2 (15.2)	
**Undergo annual ovarian screening**		0.38
**No**	38.2 (23.3)	
**Yes**	33.3 (18.1)	
**IES score**		0.77
≤25	35.5 (20.9)	
>25	35.1 (19.7)	
**Time since genetic testing (months)**		0.21
≤ 36	40.9 (21.5)	
> 36	33.2 (19.9)	
**Perceived Risk of Ovarian Cancer**		0.38
<25%	33.0 (20.9)	
≥ 25%	36.0 (20.5)	
**Planning on RRSO in future**		0.64
No	34.0 (20.6)	
Yes	36.0 (20.6)	

**Table 3:** Multivariable regression for predicting decisional conflict scores, *n* = 82

Predictor	β (95% C.I)	*p*-value
**Age (per year increase)**	− 0.54 (-1.22, 0.14)	0.12
**Education**		
No university degree	0.00	
University degree	11.40 (0.59, 22.20)	**0.039**
**Marital Status**		
Single/ Divorced/separated	0.00	
Married/common-law	-4.27 (-17.31, 8.77)	0.52
**Household income**		
Less than $80,000	0.00	
$80,000 or more	-10.03 (-21.79, 1.73)	0.09
**Race**		
White non-Hispanic	0.00	
Non-White race	11.12 (0.66, 21.57)	**0.037**
**Have children**		
No	0.00	
Yes	22.89 (10.07, 35.71)	**< 0.001**
**Planning to have children**		
No	0.00	
Yes	9.82 (-3.65, 23.29)	0.15
**BRCA pathogenic variant**		
* BRCA1*	0.00	
* BRCA2*	− 0.46 (-9.59, 8.66)	0.91
**Family History Ovarian Cancer**		
No	0.00	
Yes	7.84 (-3.94, 19.63)	0.19
**Undergo annual ovarian screening**		
No	0.00	
Yes	-3.97 (-12.64, 4.69)	0.36
**IES score**	− 0.02 (-0.30, 0.26)	0.90
**Time since genetic testing (months)**		
* ≤* 36	0.00	
> 36	-13.14 (-23.27, -2.99)	**0.012**
**Perceived Risk of Ovarian Cancer**		
<25%	0.00	
* ≥* 25%	3.64 (-7.33, 14.62)	0.51
**Planning on RRSO in future**		
No	0.00	
Yes	-2.00 (-12.87, 8.87)	0.72

## Discussion

International guidelines recommend that women with a *BRCA1* or *BRCA2* pathogenic variant (PV) undergo risk reducing salpingo-oophorectomy (RRSO) to manage the increased risk of ovarian cancer, and decrease the risk of all-cause mortality [4]. For some women, the decision to have this surgery is difficult. In the current study, we have reported on decisional conflict in women with a *BRCA1* or *BRCA2* PV who had not elected for RRSO at least one year after receiving genetic test results. Overall, 41% of women had high levels of decisional conflict related to RRSO. Higher levels of decisional conflict were associated with non-White race, having children, university education, and less than three years since receipt of genetic test results.

Decisional conflict is an important determinant of decision making, and is indicative of the level of comfort or uncertainty an individual feels in making a decision. In the current study, women with a *BRCA1* or *BRCA2* PV who had not elected for RRSO had a mean DCS score of 35.4, at an average of 80.2 months since receiving genetic test results. Overall, 41% of the cohort had a DCS score greater than 37.5, which is associated with delay of decision [11]. The mean DCS score that we have reported is similar to other reports of women with a *BRCA1* or *BRCA2* PV making cancer risk reduction decisions [13, 14]. Although there have been no reports of decisional conflict in women making decisions about RRSO, it has been reported that when making decisions related to risk-reducing mastectomy, women with a *BRCA1* or *BRCA2* PV with lower levels of decisional conflict were more likely to elect for risk-reducding mastectomy [15]. The high level of decisional conflict related to RRSO in our study highlights the difficulty that many women experience when making a decision about cancer risk reduction, and may explain why uptake of RRSO is less than 70% [6].

Decisional conflict related to health decisions has been evaluated for diverse decisions. In a recent systematic review that included 253 studies evaluating decisional conflict related to various health decisions, individuals making cancer prevention decisions had some of the highest decisional conflict scores, with mean scores just slightly lower than making end-of-life decisions [16]. This highlights, and supports the current study findings, that for women who are at high-risk of developing breast and ovarian cancers, making decisions about cancer prevention may be challenging. It is therefore important that follow-up counselling, including an assessment of barriers and support in helping overcome barriers, and/or interventions after receipt of genetic test results are available to high-risk women making decisions about cancer prevention.

Previous research has reported that uptake of RRSO is significantly lower in non-Hispanic Blacks compared to non-Hispanic Whites [17, 18]. In a population-based American study, Cragun et al. reported that uptake of RRSO was 28.1% in Black women with a *BRCA1* or *BRCA2* PV compared to 76.6% in non-Hispanic Whites [17]. More recently, in a large American study that reported on uptake of risk-reducing surgery in women with a *BRCA* PV from a military healthcare system where all patients were insured, uptake of RRSO was twice as high in non-Hispanic Whites compared to non-Hispanic Blacks [19]. Most research that has examined decisional conflict has not examined the association of race and ethnicity and decisional conflict. In previous studies examining decisional conflict related to treatment decisions in parents with children with life-limiting disease [20] and in adults with diabetes [21], higher decisional conflict has been observed in non-Whites, compared to Whites. This has been attributed to mistrust and miscommunication with the healthcare system. To our knowledge, this is the first study to evaluate the association of self-reported race with decisional conflict in women with a *BRCA1* or *BRCA2* PV. We have reported that women with non-White race had significantly higher levels of decisional conflict compared to non-Hispanic Whites. This observation highlights the importance of collecting race data, and developing culturally appropriate interventions that are specific to populations.

Psychosocial functioning after genetic testing has been examined in the past, however, there is limited evidence on how various psychosocial outcomes are associated with decisional conflict regarding cancer risk management options. In the current study, cancer-related distress did not predict decisional conflict. Women who had more intrusive thoughts or avoidance related to their increased ovarian cancer risk, did not have higher levels of decisional conflict about RRSO. This is consistent with results from Rini et al. (2009), which reported that situation-specific domains of distress were not predictive of decisional conflict in women with inconclusive genetic test results considering risk reducing mastectomy [22]. However, in other patient populations including those considering breast reconstruction at the time of breast cancer diagnosis, a weak association between anxiety and decisional conflict has been reported [23]. This suggests that although women may need psychosocial support in the period following receipt of genetic test results, these support interventions may not be effective in supporting decision-making. Interventions that target decisional conflict are needed for this patient population.

We have previously reported on a theory-based behavioural intervention for women with a PV in *BRCA1* or *BRCA2* who had not elected for RRSO [9]. The intervention incorporated risk communication and behaviour change approaches designed to: [1] raise awareness of threats about *BRCA*-associated breast and ovarian cancers; [2] raise awareness of the benefits of RRSO; and [3] address barriers to help increase motivation and self-efficacy to elect for the preventive surgery. For women randomized to the intervention arm, at one year after receipt of the intervetion, decisional conflict was significantly lower than for women in the control arm who received usual care. At two years, uptake of RRSO was signficantly higher for those who received the intervention compared to those who received usual care. These results suggest that decisional conflict may play an important role in decision making related to RRSO. With the use of the psychoeducational intervention, decisional conflict decreased and subsequent uptake of RRSO increased. The current study adds to our understanding of decisional conflict in this patient population, and we now have evidence of the factors that are associated with high decisional conflict related to RRSO after genetic testing.

Perceived risk of cancer has been shown to be associated with health behaviours, including uptake of risk-reducing mastectomy [24]. Previous research that examined decisional conflict related to cancer risk reduction in women who had received inconclusive *BRCA1* or *BRCA2* genetic test results reported that one of the most significant predictors of decisional conflict was perceived risk of cancer [22]. Women with higher perceived risk were less likely to have made decisions about cancer risk management. However, in the current study, there was no association between personal ovarian cancer risk estimation and decisional conflict. All of the women included in this study had undergone genetic counselling which has been shown to have beneficial effects on cancer risk perception [25], which may have impacted cancer risk perception. It will be important to further examine the association of decisional conflict related to RRSO with cancer risk perception, in addition to exploring women’s reasons for declining or delaying RRSO.

There are limitations to this study, including that participants were only recruited from North America, and the results may not be generalizable internationally. In addition, the women included in this study self-selected to be involved in a research study and may not be representative of the population of women with a *BRCA1* or *BRCA2* PV. This study was cross-sectional and did not evaluate changes in decisional conflict over time. However, we did observe that decisional conflict did decrease by time since genetic testing and this may be important to consider in the short and long-term management of women with a *BRCA* mutation. Prospective data to evaluate changes in decisional conflict over time is needed. Finally, the study population was primarily non-Hispanic White thereby limiting the generalizability of our findings, however, we did observe that decisional conflict was significiantly higher in non-Whites. .

Overall, this study provides evidence that many women who have been identified with a *BRCA* PV who have not elected for the recommended RRSO were experiencing high-levels of decisional conflict related to the decision to have RRSO. Higher levels of decisional conflict were associated with non-White race, having children, higher levels of education, and shorter time since the receipt of genetic test results. Decisional conflict was not associated with cancer risk perception or cancer-related distress, highlighting that interventions to help high-risk women make decisions about RRSO should specifically target decisional conflict and not psychosocial functioning.

## Data Availability

The datasets generated and/or analysed during the current study are not publicly available due not having consent from participants to share data but are available from the corresponding author on reasonable request.

## References

[1] Kuchenbaecker KB, Hopper JL, Barnes DR, Phillips KA, Mooij TM, Roos-Blom MJ, et al. Risks of breast, Ovarian, and contralateral breast cancer for BRCA1 and BRCA2 mutation carriers. JAMA. 2017;317(23):2402–16. 28632866 10.1001/jama.2017.7112

[2] Finch AP, Lubinski J, Moller P, Singer CF, Karlan B, Senter L, et al. Impact of oophorectomy on cancer incidence and mortality in women with a BRCA1 or BRCA2 mutation. J Clin Oncology: Official J Am Soc Clin Oncol. 2014;32(15):1547–53. 10.1200/JCO.2013.53.2820 PMC4026578 24567435

[3] Gronwald J, Lubinski J, Huzarski T, Cybulski C, Menkiszak J, Siolek M, et al. A comparison of ovarian cancer mortality in women with BRCA1 mutations undergoing annual ultrasound screening or preventive oophorectomy. Gynecol Oncol. 2019;155(2):270–4. 31500890 10.1016/j.ygyno.2019.08.034

[4] NCCN Clinical Practice Guidelines in Oncology. Genetic/Familial High-Risk assessment: Breast, ovarian. Pancreatic and Prostate National Comprehensive Cancer Network; 2024. 10.6004/jnccn.2021.0001 33406487

[5] Kotsopoulos J, Gronwald J, Huzarski T, Moller P, Pal T, McCuaig JM, et al. Bilateral oophorectomy and All-Cause mortality in women with BRCA1 and BRCA2 sequence variations. JAMA Oncol. 2024;10(4):484–92. 38421677 10.1001/jamaoncol.2023.6937 PMC10905374

[6] Metcalfe K, Eisen A, Senter L, Armel S, Bordeleau L, Meschino WS, et al. International trends in the uptake of cancer risk reduction strategies in women with a BRCA1 or BRCA2 mutation. Br J Cancer. 2019;121(1):15–21. 30971774 10.1038/s41416-019-0446-1 PMC6738089

[7] Thompson-Leduc P, Turcotte S, Labrecque M, Legare F. Prevalence of clinically significant decisional conflict: an analysis of five studies on decision-making in primary care. BMJ Open. 2016;6(6):e011490. 27354076 10.1136/bmjopen-2016-011490 PMC4932317

[8] O’Connor A, Sun Q, Laupacis A, Man Son Hing M, Dodin S, Felman-Stewart D, et al. editors. Predicting downstream effects of decisional conflict: Meta-analysis of the decisional conflict scale. 3rd International Shared Decision Making Conference; 2005; Ottawa, ON.

[9] Metcalfe KA, Pal T, Narod SA, Armel S, Shickh S, Buckley K, et al. Theory-based behavior change intervention to increase uptake of risk-reducing salpingo-oophorectomy in women with a BRCA1 or BRCA2 pathogenic variant: the PREVENT randomized controlled trial. Cancer Med. 2023;12(17):18246–57. 37602539 10.1002/cam4.6417 PMC10524042

[10] O’Connor AM. Validation of a decisional conflict scale. Med Decis Mak. 1995;15(1):25–30. 10.1177/0272989X9501500105 7898294

[11] O’Connor A. User manual: decisional conflict scale. Ottawa Health Research Institute; 1993. http://decisionaid.ohri.ca/docs/develop/User_Manuals/UM_decisional_conflict.pdf. (updated 2010).

[12] Horowitz M, Wilner N, Alvarez W. Impact of event scale: A measure of subjective stress. Psychosom Med. 1979;41(3):209–18. 472086 10.1097/00006842-197905000-00004

[13] Metcalfe KA, Dennis CL, Poll A, Armel S, Demsky R, Carlsson L, et al. Effect of decision aid for breast cancer prevention on decisional conflict in women with a BRCA1 or BRCA2 mutation: a multisite, randomized, controlled trial. Genet Med. 2017;19(3):330–6. 27584910 10.1038/gim.2016.108

[14] Lautz Z, Kautz-Freimuth S, Shukri A, Redaelli M, Rhiem K, Schmutzler R, et al. Predictors of knowledge and knowledge gain after decision aid use among women with BRCA1/2 pathogenic variants. Patient Educ Couns. 2024;124:108248. 38513456 10.1016/j.pec.2024.108248

[15] Dick J, Tuchler A, Bredart A, Vitinius F, Wassermann K, Rhiem K, et al. Psychological factors and the uptake of preventative measures in BRCA1/2 pathogenic variant carriers: results of a prospective cohort study. Hered Cancer Clin Pract. 2022;20(1):38. 36536421 10.1186/s13053-022-00244-y PMC9761978

[16] Garvelink MM, Boland L, Klein K, Nguyen DV, Menear M, Bekker HL, et al. Decisional conflict scale findings among patients and surrogates making health decisions: part II of an anniversary review. Med Decis Mak. 2019;39(4):315–26. 10.1177/0272989X19851346 31142205

[17] Cragun D, Weidner A, Lewis C, Bonner D, Kim J, Vadaparampil ST, et al. Racial disparities in BRCA testing and cancer risk management across a population-based sample of young breast cancer survivors. Cancer. 2017;123(13):2497–505. 28182268 10.1002/cncr.30621 PMC5474124

[18] Chapman-Davis E, Zhou ZN, Fields JC, Frey MK, Jordan B, Sapra KJ, et al. Racial and ethnic disparities in genetic testing at a hereditary breast and ovarian cancer center. J Gen Intern Med. 2021;36(1):35–42. 32720237 10.1007/s11606-020-06064-x PMC7859010

[19] Vargason AB, Turner CE, Shriver CD, Ellsworth RE. Genetic testing in Non-Hispanic black women with breast cancer treated within an equal-access healthcare system. Genet Med. 2022;24(1):232–7. 34906450 10.1016/j.gim.2021.08.002

[20] Knapp C, Sberna-Hinojosa M, Baron-Lee J, Curtis C, Huang IC. Does decisional conflict differ across race and ethnicity groups? A study of parents whose children have a life-threatening illness. J Palliat Med. 2014;17(5):559–67. 24720434 10.1089/jpm.2013.0604 PMC4912691

[21] Peek ME, Gorawara-Bhat R, Quinn MT, Odoms-Young A, Wilson SC, Chin MH. Patient trust in physicians and shared decision-making among African-Americans with diabetes. Health Commun. 2013;28(6):616–23. 23050731 10.1080/10410236.2012.710873 PMC3766485

[22] Rini C, O’Neill SC, Valdimarsdottir H, Goldsmith RE, Jandorf L, Brown K, et al. Cognitive and emotional factors predicting decisional conflict among high-risk breast cancer survivors who receive uninformative BRCA1/2 results. Health Psychol. 2009;28(5):569–78. 19751083 10.1037/a0015205 PMC3510002

[23] Ter Stege JA, Oldenburg HSA, Woerdeman LAE, Witkamp AJ, Kieffer JM, van Huizum MA, et al. Decisional conflict in breast cancer patients considering immediate breast reconstruction. Breast. 2021;55:91–7. 33387811 10.1016/j.breast.2020.12.001 PMC7779862

[24] Metcalfe KA, Narod SA. Breast cancer risk perception among women who have undergone prophylactic bilateral mastectomy. J Natl Cancer Inst. 2002;94(20):1564–9. 12381709 10.1093/jnci/94.20.1564

[25] Culver JO, Bertsch NL, Kurz RN, Cheng LL, Pritzlaff M, Rao SK, et al. Systematic evidence review and meta-analysis of outcomes associated with cancer genetic counseling. Genet Med. 2024;26(1):100980. 37688462 10.1016/j.gim.2023.100980 PMC11981685

